# Separate Calibration of Johnson–Cook Model for Static and Dynamic Compression of a DNAN-Based Melt-Cast Explosive

**DOI:** 10.3390/ma15175931

**Published:** 2022-08-27

**Authors:** Hanfei Xie, Xiangrong Zhang, Feichao Miao, Tao Jiang, Yingzhong Zhu, Xinxin Wu, Lin Zhou

**Affiliations:** 1 State Key Laboratory of Explosion Science and Technology, Beijing Institute of Technology, Beijing 100081, China; 2 School of Chemical Engineering, Anhui University of Science and Technology, Huainan 232001, China; 3 Chongqing Hongyu Precision Industry Group Co., Ltd., Chongqing 402760, China

**Keywords:** static compression, dynamic compression, strain rate, temperature, Johnson–Cook model, DNAN-based melt-cast explosive

## Abstract

When describing the relation between the flow stress and plastic strain of a material under a wide range of strain rates and temperatures, the original Johnson–Cook model generally requires a complicated modification, resulting in a loss of simplicity and clear physical interpretation. In this paper, without modification, the original Johnson–Cook model was calibrated separately for the static and dynamic compression of a DNAN-based melt-cast explosive. The stress–strain curves for static and dynamic compression of this explosive were experimentally measured with a universal testing machine and a split-Hopkinson pressure bar, respectively. Based on the stress–strain curves, the flow stress vs. plastic strain data were extracted and used to calibrate the Johnson–Cook model. The calibration process is described. The parameters for the strain term, strain rate term, and temperature term were fitted sequentially. One set of model parameters was not able to fully describe the relationship between flow stress and plastic strain for both the static and dynamic compression of the DNAN-based melt-cast explosive. Two sets of model parameters were separately calibrated and compared for the static and dynamic compression of this explosive. The effects of the adiabatic temperature rise and the definition of the yield point on this calibration were also investigated.

## 1. Introduction

Performance and safety are the two most important aspects of an explosive. Moreover, its mechanical properties are also crucial [1,2,3,4]. It is usually believed that improving the mechanical properties can enhance safety [5,6]. Thus, if an explosive is unable to resist external mechanical stimuli, defects such as cracks may develop inside an explosive charge, probably resulting in the hot-spot initiation of such a heterogeneous explosive [7,8,9]. Therefore, chemical additives or binders are often incorporated into the formulation of an explosive to improve its mechanical properties [10,11,12].

As typical mechanical properties of materials, regardless of whether they are inert or energetic, the stress–strain curves are widely investigated [13]. These data can be measured under tension or compression, and the test samples can be loaded statically or dynamically. Generally, typical stress–strain curves depend on the strain rate and temperature [14,15], i.e., the stress (σ) is a function of strain (ε), strain rate ($\dot{\varepsilon }$), and temperature (*T*).

Due to its simplicity and its use of the three factors (ε, $\dot{\varepsilon }$, and *T*), the Johnson–Cook (JC) model has been widely used for constitutive modeling based on measured stress–strain curves at different strain rates and temperatures [16,17]. This model was originally used to describe the stress–strain characteristics of metallic materials [16,18,19] and was then extended to non-metallic materials [20,21,22]. Moreover, the original JC model has been extensively modified so that it can accommodate other materials under different mechanical conditions [23,24,25,26,27].

2,4-dinitroanisole (DNAN)-based melt-cast explosives have demonstrated the characteristics of equivalent or improved extremely insensitive detonative substances compared with trinitrotoluene (TNT)-based melt-cast explosives, while maintaining the performance requirements of explosives [28,29]. DNAN-based melt-cast explosives are, therefore, widely used worldwide instead of TNT-based melt-cast explosives. However, their mechanical properties, especially their stress–strain characteristics, have not been fully investigated, which is limiting their further application. Although Li et al. have investigated the constitutive modeling of a DNAN-based melt-cast explosive based on measured stress–strain curves [22], this investigation considered those properties only at room temperature and for a limited range of strain rates.

We, thus, present herein a systematic study of the constitutive modeling (using a JC model) of a DNAN-based melt-cast explosive. The stress–strain curves under both static and dynamic conditions were measured with a universal testing machine (UTM) and split-Hopkinson pressure bar (SHPB), respectively. Furthermore, the effects of different strain rates were investigated for static and dynamic conditions. The effect of temperature on the stress–strain characteristics of the DNAN-based melt-cast explosive was also investigated. The fittings of the measured stress–strain curves are compared and discussed for both static and dynamic compression.

## 2. Experiments

### 2.1. Material

**Figure 1:**
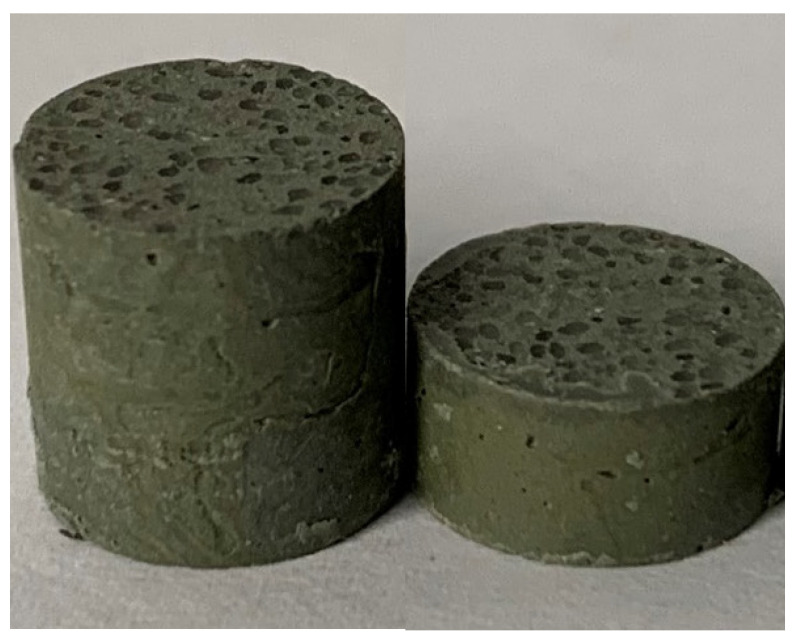
Photograph of test samples.

The melt-cast explosive used in this study consists of 25 wt% DNAN, 36 wt% RDX (cyclotrimethylenetrinitramine), 31 wt% Al (aluminum), and 8 wt% additives. First, the DNAN was melted at 100 ${}^{{}^{\circ}}C$ in a double-jacketed stainless-steel kettle equipped with a stirrer. Second, the RDX and Al solids were added incrementally to the molten DNAN and stirred at a rate of 200–300 rev/min. After adding the solid to the DNAN, the mixture was stirred for 15 min at a rate of 500 rev/min to ensure uniform mixing and to eliminate any solid agglomerates. Finally, the uniformly mixed suspension of the DNAN-based melt-cast explosive was slowly poured into a mold and cooled down to room temperature. For both static and dynamic measurements, the diameter of the cylindrical samples of this explosive were 10 mm, whereas the heights were 10 mm or 5 mm for static and dynamic measurements, respectively. Figure 1 is a photograph of two typical samples.

### 2.2. Method

Based on the magnitude of the strain rate, measured stress–strain curves are usually divided into two categories: static or dynamic. However, the dividing line is generally not strict. In this study, we assume that static measurements correspond to stress–strain curves where the order of magnitude of the strain rate $\dot{\varepsilon }\leq 10^{-1}\;s^{-1}$, and that dynamic measurements are for strain rates $\dot{\varepsilon }\geq 10^{1}\;s^{-1}$. Stress–strain curves where the order of magnitude of the strain rate is $10^{-1}\;s^{-1}<\dot{\varepsilon }<10^{1}\;s^{-1}$ are outside our present investigation and therefore, not considered. As mentioned in Section 1, stress–strain measurements with strain rates $\dot{\varepsilon }\leq 10^{-1}\;s^{-1}$ and $\dot{\varepsilon }\geq 10^{1}\;s^{-1}$ were measured with a UTM and SHPB, respectively.

#### 2.2.1. Static Compression

Samples of the DNAN-based melt-cast explosive were statically compressed by the UTM (Instron 5965, Norwood, MA, USA), and the corresponding stress and strain data under a given strain rate were obtained from the built-in force and displacement sensors. However, since we wished to assess the effect of temperature on the stress–strain characteristics of this explosive, a temperature control system was installed on this original UTM, as described in Section 2.2.2.

#### 2.2.2. Dynamic Compression

The dynamic compression of a material at high strain rate is generally measured by a SHPB. The main components are an impact-loading system, a signal-measuring system, and a data-processing system [30,31]. The impact-loading system has three pressure bars: a strike bar, an input bar, and an output bar. The signal-measuring system independently measures the velocity of the strike bar and the strain waves on both the input and output bars. The data-processing system analyzes the measured signals and obtains the stress–strain curve of a sample placed between the input and output bars. However, since the present samples were made from the DNAN-based melt-cast explosive, which has both low mechanical strength and low wave impedance, the impact-loading system of the SHPB had to be specially designed.

Generally, the wave impedances of both the pressure bars and the sample should be matched. Compared to the wave impedance of the steels commonly used as pressure bars, the wave impedance of the DNAN-based melt-cast explosive is considerably lower. To minimize the effect of a wave-impedance mismatch [32], the aluminum alloy LC4 was used instead for the pressure bars in this study. Moreover, due to the low mechanical strength of this explosive, a pulse shaper (made of copper) was attached to the surface of the strike bar to prevent the failure of the explosive sample before the stress balance inside the sample was reached [33].

**Figure 2:**
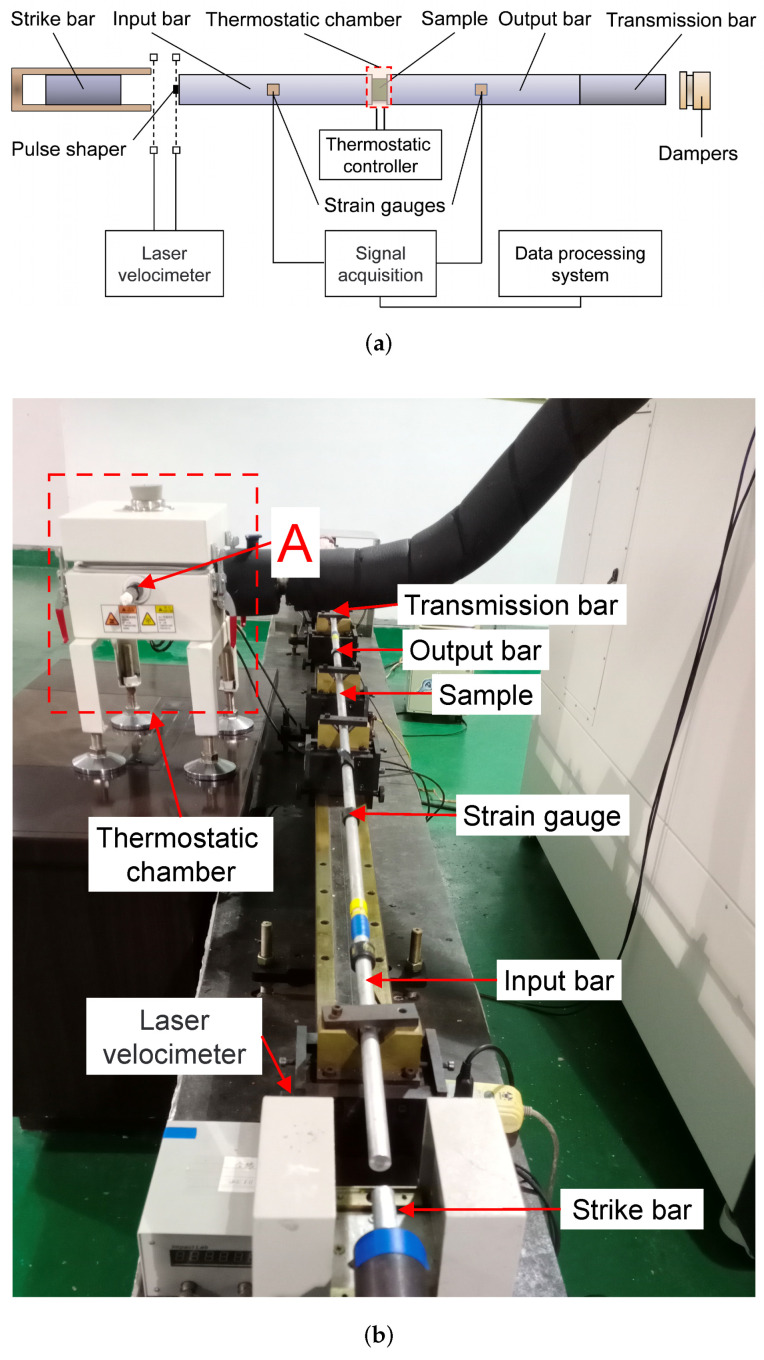
SHPB experimental setup. (**a**) schematic; (**b**) photograph.

Moreover, we wanted to investigate the effect of temperature on the static and dynamic compression of this DNAN-based melt-cast explosive. Therefore, a temperature-control system was installed that had a thermostatic controller. There was also a thermostatic chamber, which had to meet two requirements: (1) it must be able to heat or cool samples placed inside it to a stable temperature and (2) it should be able to resist a blast wave resulting from the sample accidentally exploding. Moreover, the thermostatic chamber is movable, which is necessary for a SHPB. As shown in Figure 2, before and after each experiment, the thermostatic chamber was detached from the pressure bars and placed on them during an experiment. The input and output bars go through the front (position A) and back (not shown) faces of the thermostatic chamber, respectively.

The experimental procedures had the following main steps:

1. The sample was fixed at the interfaces between the input and output bars. The temperature inside the thermostatic chamber was set to a given value. Then, the sample inside the thermostatic chamber was heated or cooled for at least 10 min to ensure that there was no temperature difference between the sample and the air inside the thermostatic chamber.
2. The strike bar was driven so that it impacted the input bar at a certain velocity. The incident and the reflected strain waves at the interface between the input bar and the sample were measured by one strain gauge, while the transmitted wave was similarly measured at the interface between the sample and the output bar.
3. The dynamic stress–strain characteristics of the DNAN-based melt-cast explosive were obtained by analyzing the measured strain wave signals. The strain rate was also calculated.

### 2.3. Results

**Figure 3:**
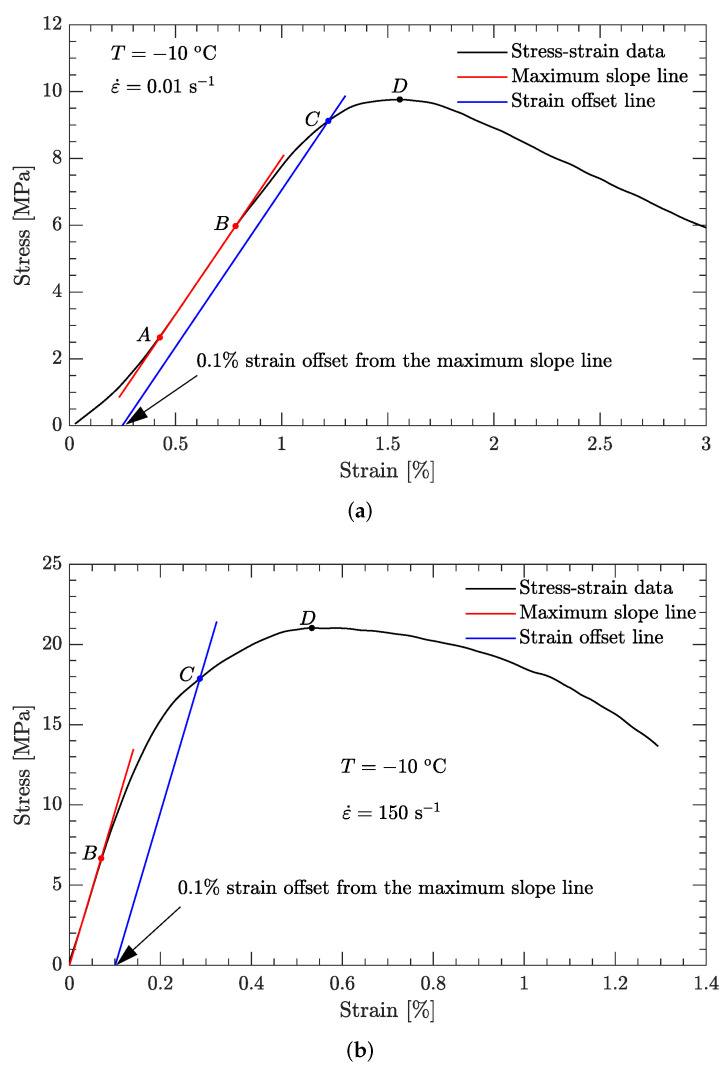
Typical stress–strain curves for both static and dynamic compression. (**a**) Static compression; (**b**) dynamic compression.

Typical stress–strain curves for both static and dynamic compression are shown in Figure 3. On the whole, both curves rise from the origin, reach a maximum, and then gradually decrease. However, for the static compression, the maximum slope of this curve is not near the origin; rather, it is roughly in the middle of the rising curve (the approximately straight line segment AB in Figure 3a). The initial region (curve segment 0A in Figure 3a) may be due to the machine compliance/closing gaps [34]. Compared to static compression, the maximum slope of the dynamic compression curve is near the origin, after which the gradient of this curve gradually decreases.

Note that engineering stress and strain are shown in Figure 3. The true stress and strain are physically reasonable and can also be used. These can be expressed in terms of the engineering stress and strain as follows [35]:

(1) $$\begin{array}{cc} \sigma_{t} & =\sigma_{e}\left(1+\varepsilon_{e}\right) \end{array}$$

(2) $$\begin{array}{cc} \varepsilon_{t} & =\ln\left(1+\varepsilon_{e}\right) \end{array}$$

where $\sigma_{t}$ and $\varepsilon_{t}$ are the true stress and true strain, respectively, whereas $\sigma_{e}$ and $\varepsilon_{e}$ are the engineering stress and engineering strain, respectively. Since the measured deformation of this DNAN-based melt-cast explosive is generally less than 3% (Figure 3), the difference between the engineering strain and the true strain is negligibly small according to Equation (2). Therefore, we assume that the engineering strain is the same as the true strain, and they are both called the strain ε. Similarly, both the engineering stress and the true stress are called the stress σ.

Based on the shape of the typical stress–strain curve in Figure 3, despite that the strain at the ultimate stress (point D in Figure 3a, for example) is generally less than 2% for both static and dynamic compression, it is reasonably assumed that plastic deformation occurred. However, the location of the yield point (where permanent deformation begins to develop) on the measured stress–strain curve is not obvious. Moreover, locating this point is somewhat arbitrary in the literature. Without validation, the 0.2% offset yield stress is widely used for many materials. However, in some cases, the yield stress derived from the strain offset of 0.2% is considerably higher than the experimentally measured value, especially for brittle materials [36,37]. Therefore, in this study, a strain offset of 0.1% was arbitrarily used to locate the yield point of the DNAN-based melt-cast explosives, and the yield point was defined as the intersection with a line parallel to the maximum slope but offset by 0.1%. This gives the points C in Figure 3.

**Figure 4:**
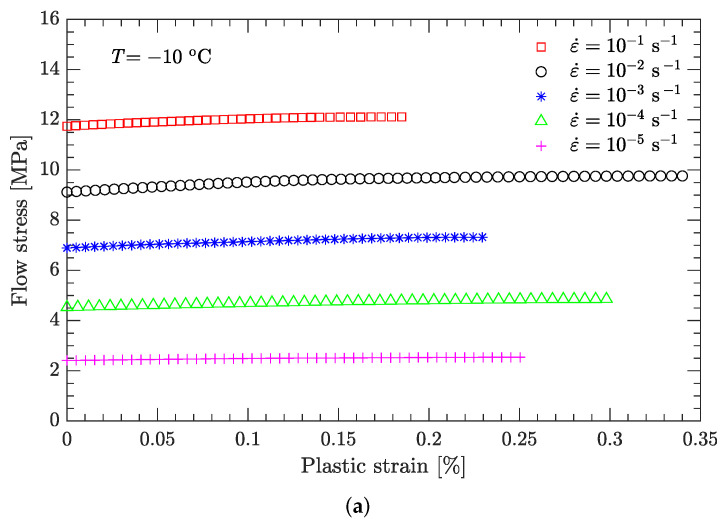

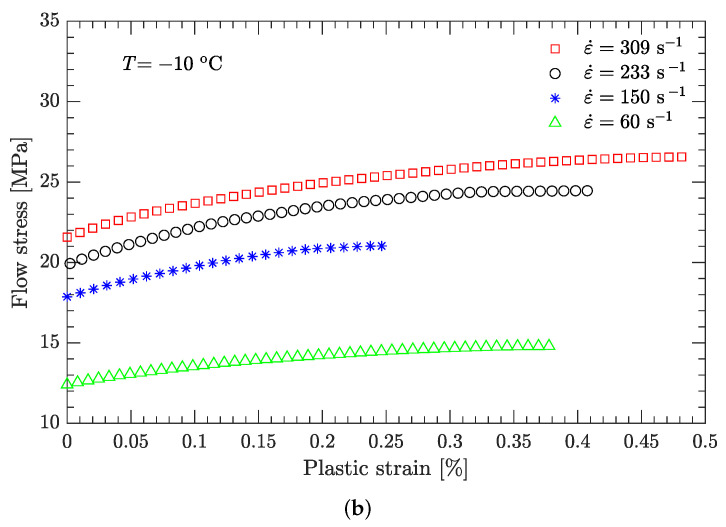
Measured flow stress vs. plastic strain at a temperature of $-10{\;}^{{}^{\circ}}C$. (**a**) Static compression; (**b**) dynamic compression.

**Figure 5:**
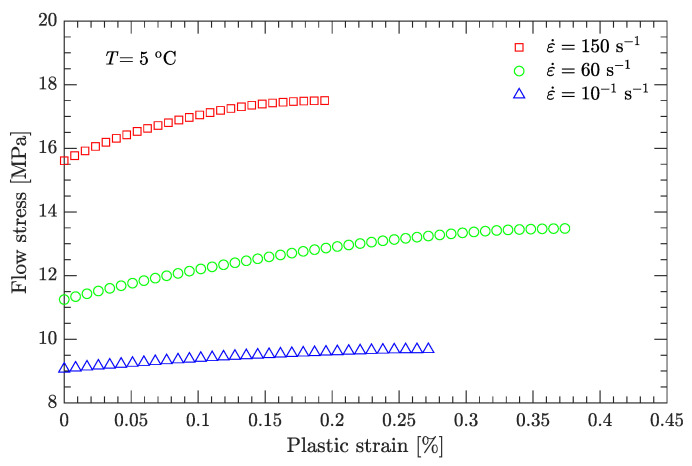
Measured flow stress vs. plastic strain at a temperature of $5{\;}^{{}^{\circ}}C$.

**Figure 6:**
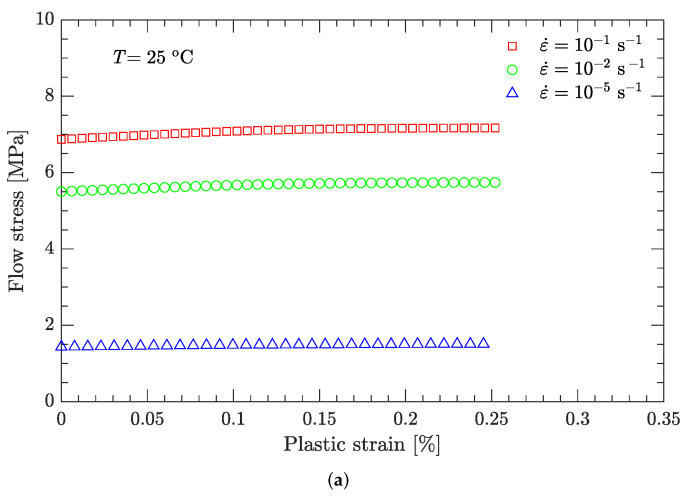

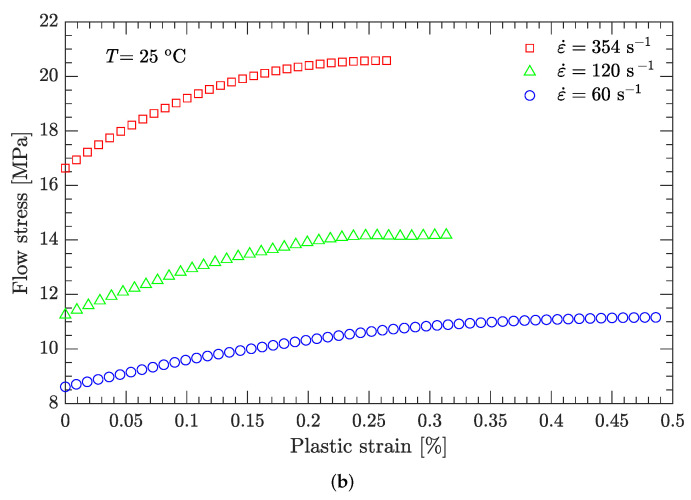
Measured flow stress vs. plastic strain at a temperature of $25{\;}^{{}^{\circ}}C$. (**a**) Static compression; (**b**) dynamic compression.

**Figure 7:**
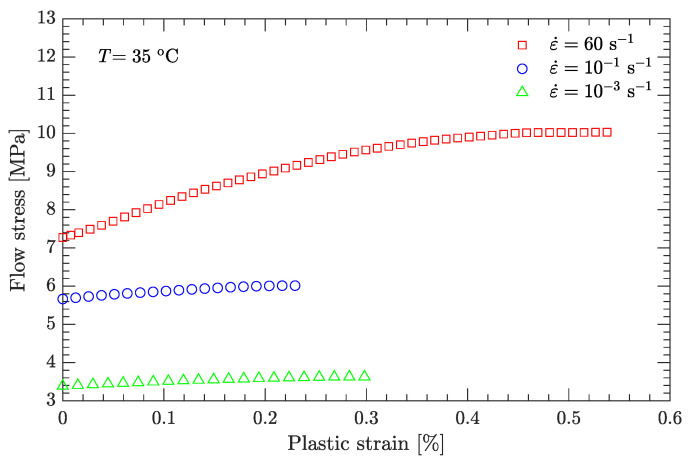
Measured flow stress vs. plastic strain at a temperature of $35{\;}^{{}^{\circ}}C$.

**Figure 8:**
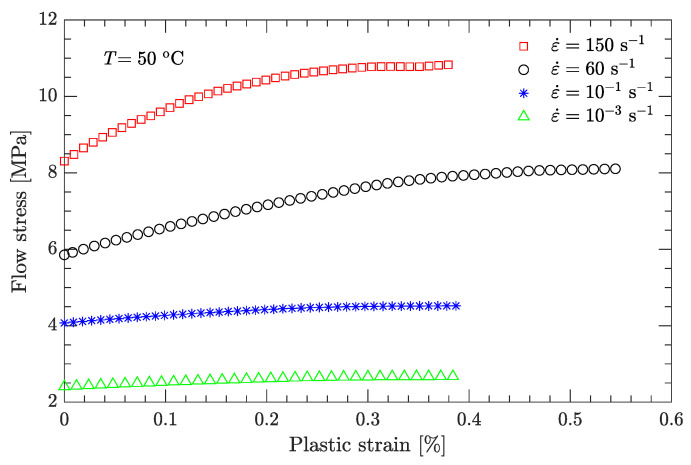
Measured flow stress vs. plastic strain at a temperature of $50{\;}^{{}^{\circ}}C$.

Using this definition of the yield point, the flow stress region, which is between the yield stress and ultimate the stress of the DNAN-based melt-cast explosive, can be extracted (curve segment CD in Figure 3a). Then, the relation between flow stress ($\sigma_{f}$) and plastic strain ($\varepsilon_{p}$) can be established (Figure 4, Figure 5, Figure 6, Figure 7 and Figure 8). Note that the plastic strain (abscissa of these figures) was calculated by subtracting the elastic portion from the strain. All three effects (strain hardening, strain rate hardening, and thermal softening) are visible in these flow stress vs. plastic strain curves of the DNAN-based melt-cast explosive, no matter whether it is undergoing static or dynamic compression.

## 3. Constitutive Modeling

As mentioned in Section 1, the measured flow stress vs. plastic strain curves of the DNAN-based melt-cast explosive are fitted with the JC model. In this section, first, we describe the procedure for fitting the parameters and then discuss the predictability of the fitted parameters. Two sets of parameters are separately fitted for static and dynamic compression. Finally, we investigate the effects of adiabatic temperature rise and the definition of yield stress on the calibration of the JC model.

### 3.1. Parameter Fitting Procedure

The classical JC constitutive model is given by [16]

(3) $$\sigma_{f}=\left(A+B\varepsilon_{p}^{n}\right)\left(1+C\ln\frac{\dot{\varepsilon }}{\dot{\varepsilon_{0}}}\right)\left[1-{\left(\frac{T-T_{r}}{T_{m}-T_{r}}\right)}^{m}\right]$$

where $\sigma_{f}$ and $\varepsilon_{p}$ are the flow stress and plastic strain, respectively. $\dot{\varepsilon }$ and ${\dot{\varepsilon }}_{0}$ are the strain rate and reference strain rate, respectively. *T* is the temperature of the sample. $T_{r}$ and $T_{m}$ are the reference temperature and melting temperature, respectively. *A*, *B*, *n*, *C*, and *m* are the parameters to be fitted. Clearly, *A* is the yield stress at the reference strain rate and reference temperature, whereas *B* and *n* are the strain hardening coefficient and exponent, respectively. *C* and *m* represent the strain rate hardening coefficient and the thermal softening exponent, respectively.

A commonly used method was adopted to fit all five parameters (*A*, *B*, *n*, *C*, and *m*) [26,27]. Parameters *A*, *B*, and *n*, which characterize the strain hardening, were first fitted at the reference strain rate and reference temperature. Then, parameter *C*, which characterizes the strain rate hardening, was fitted at the reference temperature. Finally, parameter *m*, which characterizes the thermal softening, was fitted at the reference strain rate.

#### 3.1.1. Parameters *A*, *B*, and *n*

At the reference strain rate and reference temperature, i.e., when $\dot{\varepsilon }={\dot{\varepsilon }}_{0}$ and $T=T_{r}$, the JC model of Equation (3) reduces to

(4) $$\sigma_{f}=\left(A+B\varepsilon_{p}^{n}\right).$$

The yield stress *A* can easily be read from the flow stress vs. plastic strain curve (Figure 4a) at the reference strain rate ($10^{-1}\;s^{-1}$) and reference temperature ($-10{\;}^{{}^{\circ}}C$). In this way, the value of *A* was determined to be 11.744 MPa.

To determine the values of parameters *B* and *n*, Equation (4) is transformed to

(5) $$\ln\left(\sigma_{f}-A\right)=\ln B+n\ln\varepsilon_{p}.$$

**Figure 9:**
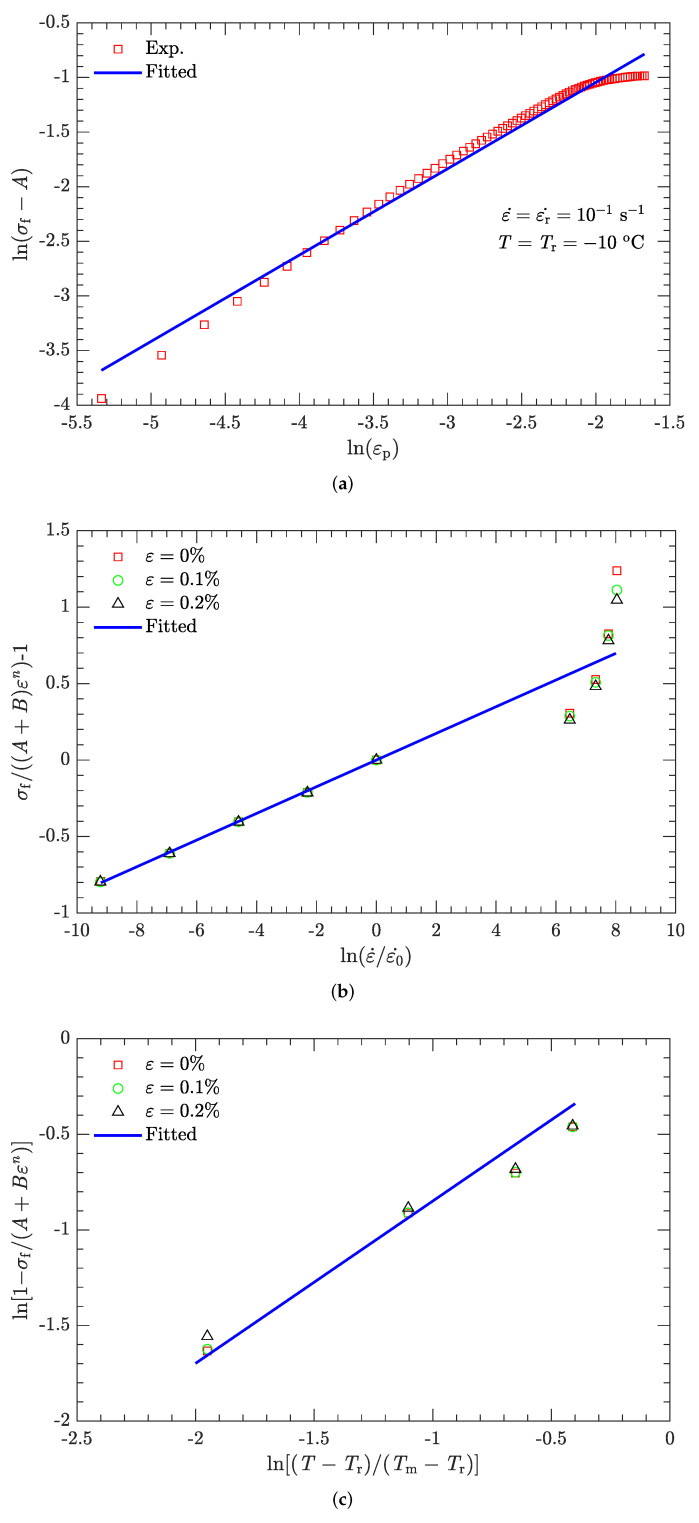
Linear regression for parameters *A*, *B*, *n*, *C*, and *m*. (**a**) Parameters *A*, *B*, and *n*; (**b**) parameter *C*; and (**c**) parameter *m*.

With *A* already determined, the values of lnB and *n* can easily be fitted by linear regression of the flow stress vs. plastic strain data (Figure 4a) at the reference strain rate ($10^{-1}\;s^{-1}$) and reference temperature ($-10{\;}^{{}^{\circ}}C$) (Figure 9a). In this way, the values of *B* and *n* were determined to be 1.708 MPa and 0.790, respectively.

#### 3.1.2. Parameter *C*

At the reference temperature, i.e., when $T=T_{r}$, the JC model of Equation (3) reduces to

(6) $$\sigma_{f}=\left(A+B\varepsilon_{p}^{n}\right)\left(1+C\ln\frac{\dot{\varepsilon }}{\dot{\varepsilon_{0}}}\right).$$

Rearranging Equation (6):

(7) $$\frac{\sigma_{f}}{A+B\varepsilon_{p}^{n}}-1=C\ln\frac{\dot{\varepsilon }}{\dot{\varepsilon_{0}}},$$

where the value of ${\dot{\varepsilon }}_{0}$ was previously specified ($10^{-1}\;s^{-1}$), and parameters *A*, *B*, and *n* were determined above. At the reference temperature ($-10{\;}^{{}^{\circ}}C$) and under a certain plastic strain, there are nine flow stress–strain rate curves (Figure 4). Therefore, as for parameters *B* and *n*, parameter *C* can also be fitted by linear regression (Figure 9b). It was determined to be 0.087. However, as indicated in Figure 9b, the coefficient of determination during the linear regression $R^{2}$ is not very high (only 0.91). This issue will be further discussed in Section 3.2.1.

#### 3.1.3. Parameter *m*

At the reference strain rate, i.e., when $\dot{\varepsilon }=\dot{\varepsilon_{0}}$, the JC model of Equation (3) reduces to

(8) $$\sigma_{f}=\left(A+B\varepsilon_{p}^{n}\right)\left[1-{\left(\frac{T-T_{r}}{T_{m}-T_{r}}\right)}^{m}\right].$$

Rearranging Equation (8):

(9) $$\ln\left(1-\frac{\sigma_{f}}{A+B\varepsilon_{p}^{n}}\right)=m\ln\frac{T-T_{r}}{T_{m}-T_{r}},$$

where the melting temperature ($T_{m}$) of the DNAN-based melt-cast explosive is $92.5{\;}^{{}^{\circ}}C$. The reference temperature $T_{r}$ was previously specified ($-10{\;}^{{}^{\circ}}C$). At the reference strain rate ($10^{-1}\;s^{-1}$) and under a certain plastic strain, there are four flow stress–strain rate curves (Figure 5, Figure 6, Figure 7 and Figure 8). Therefore, parameter *m* can be fitted in the same way as parameter *C* (Figure 9c). The value of *m* was determined to be 0.815.

### 3.2. Results and Discussion

#### 3.2.1. Predictability of the Calibrated JC Model

Since all five parameters (*A*, *B*, *n*, *C*, and *m*) in the JC model have been determined, the calibrated model can quantify the relation between flow stress and plastic strain at a given strain rate and temperature. In this section, we first compare the error between the flow stress calculated by the JC model and the measured data used to calibrate this model. Then, the calibrated JC model was used to predict the flow stress. The predictability of the calibrated JC model was investigated with the measured data not used to calibrate this model.

**Figure 10:**
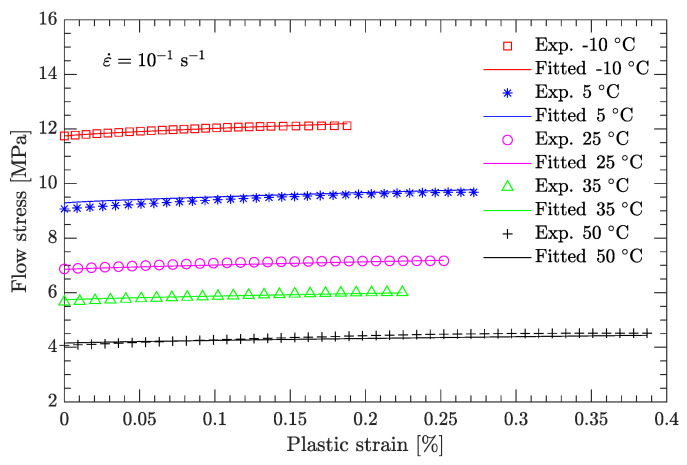
Comparison of fitted and measured flow stresses at a strain rate of $10^{-1}\;s^{-1}$.

**Figure 11:**
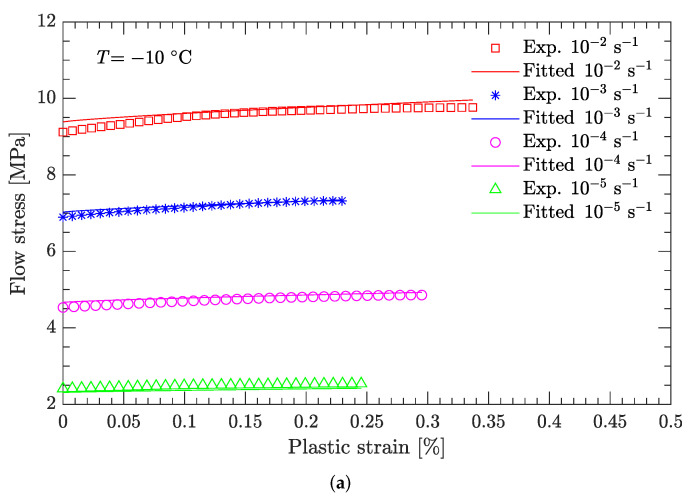

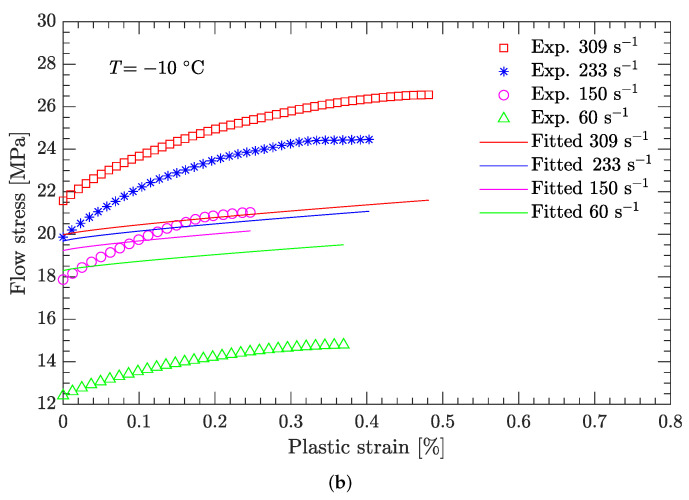
Comparison of fitted and measured flow stresses at a temperature of $-10{\;}^{{}^{\circ}}C$. (**a**) Static compression; (**b**) dynamic compression.

**Figure 12:**
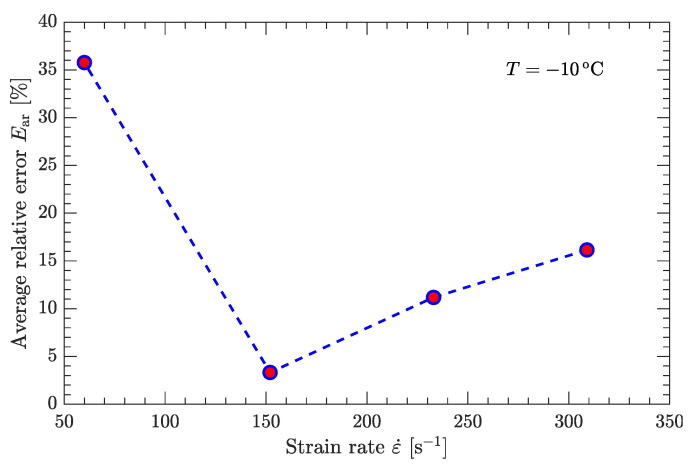
Average relative error between fitted and measured flow stresses.

Figure 10 and Figure 11 compare the calculated and experimental flow stress data at the reference strain rate and reference temperature, respectively. At the reference strain rate ($\dot{\varepsilon }=10^{-1}\;s^{-1}$), the calculated flow stress considering thermal softening agrees well with the experimental data (Figure 10), regardless of whether the temperature was high or low. In contrast, at the reference temperature ($T=-10{\;}^{{}^{\circ}}C$), the calculated flow stress considering strain rate hardening is not completely consistent with the experimental data (Figure 11). For low strain rates (static compression), the calculated flow stress agrees well with the experimental data (Figure 11a), whereas for high strain rates (dynamic compression), the deviation between the calculated and experimental flow stress is considerably large (Figure 11b). Figure 12 further examines this deviation using the average relative error, which is defined as

(10) $$E_{\mathrm{ar}}=\frac{1}{N_{t}}\sum_{i=1}^{N_{t}}\left|\frac{\sigma_{f}\left(\varepsilon_{p,i}\right)-\hat{\sigma_{f}}\left(\varepsilon_{p,i}\right)}{\hat{\sigma_{f}}\left(\varepsilon_{p,i}\right)}\right|\times 100\%,$$

where $\sigma_{f}$ and $\hat{\sigma_{f}}$ are the calculated and experimental values of the flow stress, respectively. $N_{t}$ is the number of plastic strain points ($\varepsilon_{p}$) used to calculate the average relative error ($E_{\mathrm{ar}}$). As shown in Figure 12, the maximum average relative error is larger than 35% at high strain rates, which is partially due to the relatively low value of the coefficient of determination $R^{2}$ (Figure 9c).

Note that the experimental data shown in Figure 10 and Figure 11 were used to calibrate the JC model. Since the calculated flow stress at high strain rates (dynamic compression) does not agree with the measured data used to calibrate the JC model, it was difficult to predict the flow stress from the measured data not used to calibrate the JC model. Therefore, the current JC parameter set can predict only the flow stress at low strain rates (static compression).

**Figure 13:**
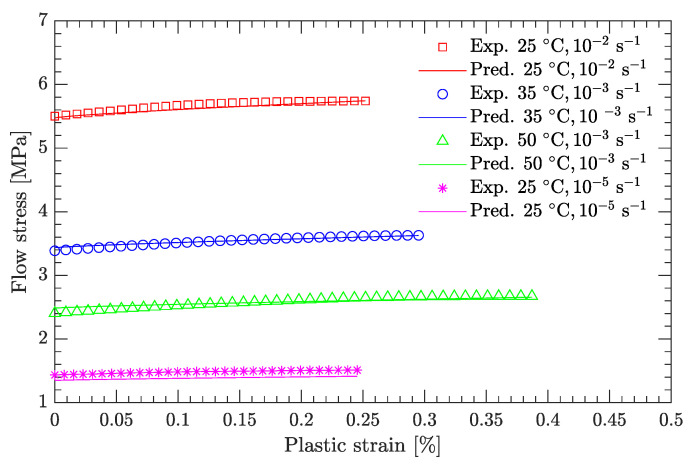
Comparison of predicted and measured flow stresses under static compression.

Figure 13 compares the flow stress predicted by the current JC parameter set with experimental data other than the reference strain rate and reference temperature. It can be seen that the predicted flow stress is in good agreement with the experimental data, demonstrating that the current JC parameter set is suitable for predicting the flow stress of the DNAN-based melt-cast explosives at low strain rates (static compression).

#### 3.2.2. Separate Parameter Sets for Static and Dynamic Compression

The current JC parameter set cannot completely predict the flow stress under both static (low strain rates) and dynamic (high strain rates) compression of the DNAN-based melt-cast explosive. A traditional method of overcoming this is to modify the original JC model so that it covers the entire strain rate range. However, such modification usually complicates the JC model, resulting in a loss of simplicity and clear physical interpretation. An alternative is to calibrate the JC model separately for static and dynamic compression.

Thus, a new JC parameter set needs to be calibrated for the flow stress under dynamic compression since the previously calibrated JC parameter set can accurately predict the flow stress under static compression. The procedure for fitting the parameters of the JC model under dynamic compression is the same as that under static compression, except that the reference strain rate is different. Compared to the reference strain rate ($0.1\;s^{-1}$) under static compression, the reference strain rate is $60\;s^{-1}$ under dynamic compression.

**Figure 14:**
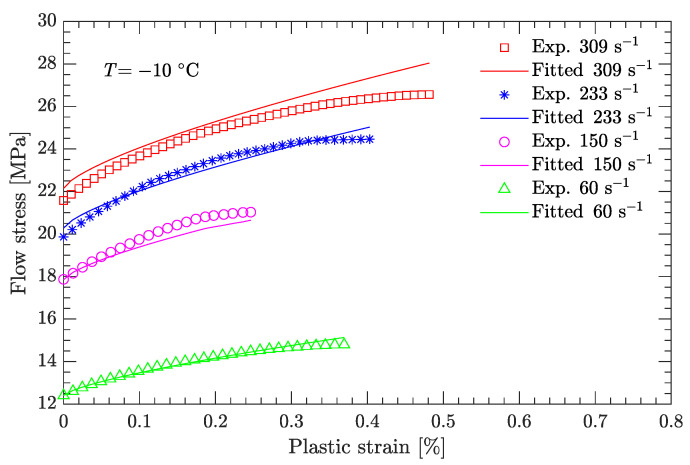
Comparison of fitted and measured flow stresses at a temperature of $-10{\;}^{{}^{\circ}}C$.

**Figure 15:**
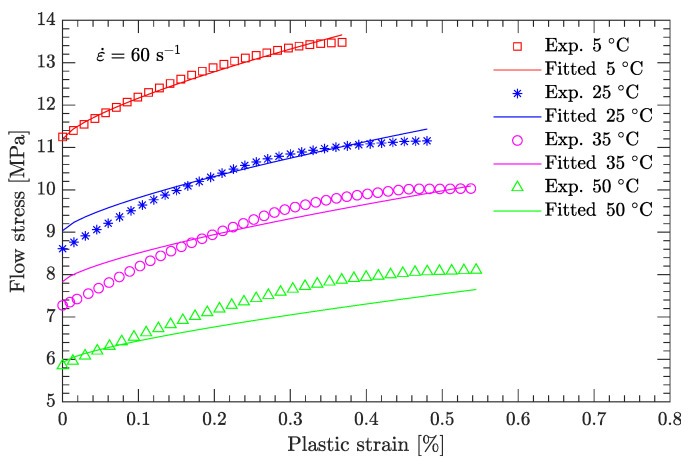
Comparison of fitted and measured flow stresses at a strain rate of $60\;s^{-1}$.

**Figure 16:**
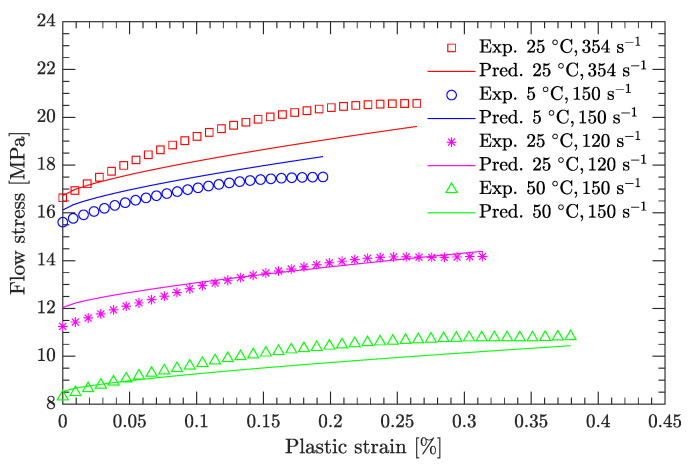
Comparison of predicted and measured flow stresses under dynamic compression.

Figure 14 and Figure 15 compare the calculated flow stress under dynamic compression with the experimental data used to calibrate the JC model. Figure 16 compares the predicted flow stress under dynamic compression with the experimental data not used to calibrate the JC model. For both cases, the average relative errors are small, with largest values of 5.45% and 4.75%, respectively, demonstrating the effectiveness of the newly calibrated JC parameter set.

The separate JC parameter sets for static and dynamic compression are listed in Table 1. Regardless of whether we are considering static or dynamic compression, the corresponding parameter set can correctly represent the effects of strain hardening, strain rate hardening, and thermal softening of the DNAN-based melt-cast explosive. However, compared to the parameter set for static compression, the effects of strain hardening (characterized by parameters *A*, *B*, and *n*), strain rate hardening (characterized by parameter *C*), and thermal softening (characterized by parameter *m*) of this explosive are more significant for dynamic compression.

#### 3.2.3. Effect of Adiabatic Temperature Rise

In the temperature term in the JC model of Equation (3), *T* is the sample temperature. Generally, this temperature is not constant, especially for dynamic compression at high strain rates. Under adiabatic conditions, the sample temperature increases due to dissipation of plastic work done [14,27,38]. Therefore, the sample temperature depends on the ambient temperature ($T_{a}$) (the air temperature inside the thermostatic chamber, Figure 2) and the adiabatic temperature rise ($∆T_{a}$), given by

(11) $$∆T_{a}=\int_{0}^{\varepsilon_{p}}\frac{\alpha \sigma_{f}}{\rho c}d\varepsilon_{p}$$

where $\rho \;\left(1800\;\mathrm{kg}/m^{3}\right)$ and $c\;\left[1045\;J/\left(\mathrm{kg}\cdot K\right)\right]$ are the density and specific heat capacity of the DNAN-based melt-cast explosive, respectively, and α is the conversion coefficient characterizing the amount of plastic work converted into heat, which is usually estimated to be 0.9 [27,38].

**Figure 17:**
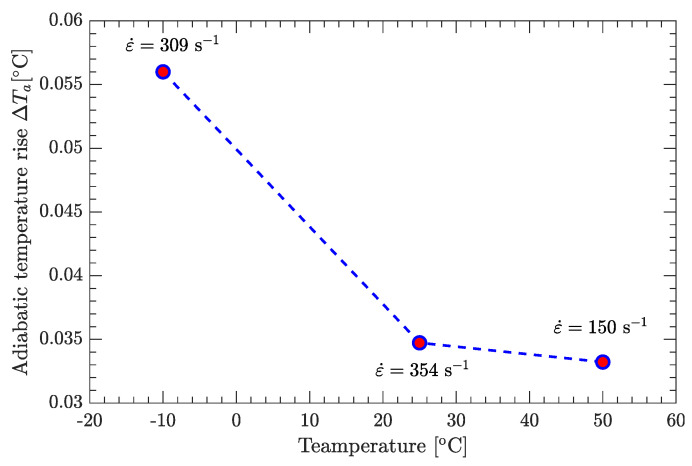
Adiabatic temperature rise due to plastic work conversion.

Figure 17 compares several values of $∆T_{a}$ at high strain rates based on the flow stress vs. plastic strain curves. The maximum of $∆T_{a}$ is only $0.056{\;}^{{}^{\circ}}C$, which is negligibly small. Therefore, in this study, the sample temperature (*T*) is assumed to be constant and equal to the ambient temperature ($T_{a}$).

#### 3.2.4. Effect of Definition of Yield Point

As mentioned in Section 2.3, a strain offset of 0.1% was used to define the yield point of the DNAN-based melt-cast explosive. Based on the offset yield stress, both the static and dynamic compression of this explosive can be described by the JC model. However, since the definition of the yield stress is somewhat arbitrary, the effect of the strain offset on the yield stress and on the calibration of the JC parameter sets for both static and dynamic compression is investigated next. Therefore, a strain offset of 0.08% was used to redefine the yield stress, and the JC model was recalibrated.

**Figure 18:**
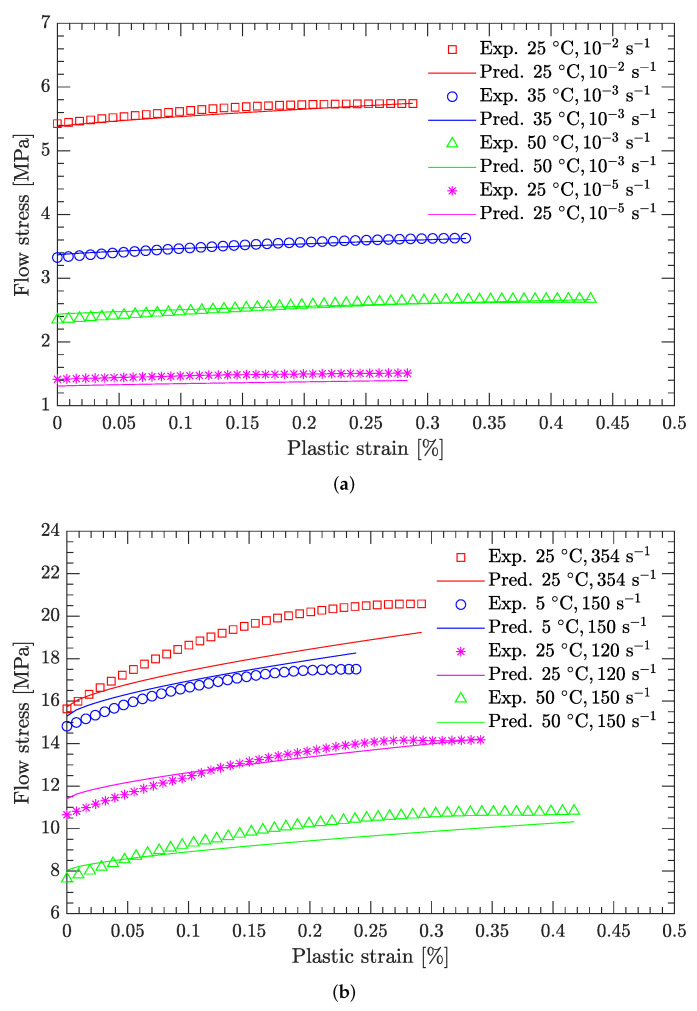
Effect of definition of yield point on the predicted flow stress. (**a**) Static compression; (**b**) dynamic compression.

After following the same fitting procedure for the parameters as used for a strain offset of 0.1%, the recalibrated JC parameter sets for a strain offset of 0.08% are listed in Table 2, and the corresponding partial prediction of the flow stress is shown in Figure 18. Obviously, compared to the yield stress (*A* in Table 1) for a strain offset of 0.1% at the reference strain rate and reference temperature, the yield stress (*A* in Table 2) for a strain offset of 0.08% is lower. However, the effect of the strain offset on the parameters *C* (characterizing the strain rate hardening) and *m* (characterizing the thermal softening) is negligibly small. The flow stress predicted by the recalibrated JC parameter set (Table 2) also agrees with the measured data (Figure 18), as it did with the previously calibrated JC parameter set, demonstrating that both static and dynamic compression of the DNAN-based melt-cast explosive can be described using the JC model, although the yield stress is defined by the strain offset.

## 4. Conclusions

The static and dynamic compression of the DNAN-based melt-cast explosive was measured with a UTM and SHPB, respectively. Both the static and dynamic stress–strain curves depend on the strain rate and temperature. In the static case, the strain rate was in the range $10^{-5}\;s^{-1}\leq \dot{\varepsilon }\leq 10^{-1}\;s^{-1}$, whereas for the dynamic case, it was in the range $60\;s^{-1}\leq \dot{\varepsilon }\leq 354\;s^{-1}$. In contrast, the temperature was always in the range $-10{\;}^{{}^{\circ}}C\leq T\leq 50{\;}^{{}^{\circ}}C$ for both static and dynamic cases. However, for both cases, the stress–strain curves represent the effects of both strain rate hardening and thermal softening. Additionally, due to the low value of the plastic strain, the difference between the engineering strain and the true strain was negligible. For the same reason, the adiabatic temperature rise during compression was also reasonably neglected.

The original JC model is simple and has a clear physical interpretation. Based on the experimentally measured stress–strain curves, this JC model was used to describe the relation between flow stress and plastic strain of the DNAN-based melt-cast explosive. However, a single parameter set for the JC model cannot accurately describe both static and dynamic compression of this explosive if the original JC model is used without complicated modification. By separately calibrating the original JC model for static compression and dynamic compression, two parameter sets were obtained.

Both parameter sets can describe the effects of strain hardening, strain rate hardening, and thermal softening of the DNAN-based melt-cast explosive. However, compared to the parameter set for static compression, the effects of strain hardening (characterized by parameters *A*, *B*, and *n*), strain-rate hardening (characterized by parameter *C*), and thermal softening (characterized by parameter *m*) of this explosive are more significant for dynamic compression.

However, in the calibration of the JC model with the flow stress vs. plastic strain curves of the DNAN-based melt-cast explosive, the determination of the yield point was somewhat arbitrary since a strain offset was used to define the yield point. This offset mainly affected the strain hardening term in the calibrated JC model parameters, whereas it had a negligible effect on strain rate hardening and thermal softening. In our future work, we will conduct more experiments, in particular, to more accurately determine the yield stress of this DNAN-based melt-cast explosive.

## Figures and Tables

**Table 1:** JC model parameter sets with 0.1% strain offset.

Compression	*A* (MPa)	*B* (MPa)	*n*	*C*	*m*
Static	11.744	1.708	0.790	0.087	0.815
Dynamic	12.404	5.574	0.717	0.479	1.213

**Table 2:** JC model parameter set with 0.08% strain offset.

Compression	*A* (MPa)	*B* (MPa)	*n*	*C*	*m*
Static	11.593	2.126	0.794	0.088	0.809
Dynamic	11.936	6.116	0.675	0.463	1.195

## Data Availability

The data presented in this study are available on request from the corresponding author.
